# Luo Tong formula attenuates retinal inflammation in diabetic rats via inhibition of the p38MAPK/NF-κB pathway

**DOI:** 10.1186/s13020-019-0284-3

**Published:** 2020-01-14

**Authors:** Bing Pang, Min Li, Jun Song, Qing-wei Li, Jia Wang, Sha Di, Xiao-lin Tong, Qing Ni

**Affiliations:** 1 Department of Endocrinology, Guang’anmen Hospital of China Academy of Chinese Medical Sciences, 6 Floors of Inpatients Building, 5 Beixiange Street, Xicheng District, Beijing, 100053 China; 2 Molecular Biology Laboratory, Guang’anmen Hospital of China Academy of Chinese Medical Sciences, Beijing, 100053 China; 3 General Department, Guang’anmen Hospital of China Academy of Chinese Medical Sciences, Beijing, 100053 China; 4 Department of Endocrinology, Guang’anmen Hospital of China Academy of Chinese Medical Sciences, Room 432, Administration Building, 5 Beixiange Street, Xicheng District, Beijing, 100053 China

**Keywords:** Diabetic retinopathy, Prevention, Luo Tong formula, Pro-inflammatory factors, NF-κB pathway, p38-MAPK pathway

## Abstract

**Background:**

Diabetic retinopathy (DR) is a serious microvascular complication of diabetes and remains the leading cause of blindness in adults. Retinal inflammation is playing a crucial role in the development of DR, and targeting inflammatory mediators is a promising strategy for controlling DR. Here, we investigated compound Chinese medicine Luo Tong formula (LTF) alleviated retinal inflammatory responses in a STZ-induced diabetic rat model.

**Methods:**

Sprague–Dawley rats were divided into four groups: control, streptozotocin-induced diabetic, LTF-treated diabetic, and calcium dobesilate (CaD)-treated diabetic rats. Blood samples were collected for blood glucose examination. Hematoxylin–eosin and periodic acid-Schiff staining were conducted for light microscopy observations. Retinal cell apoptosis was detected using the TUNEL assay. Proteins expression was quantified by Western blotting and/or immunohistochemistry, and gene expression was assessed by real-time PCR.

**Results:**

Diabetic rats showed significant increases in the expression of tumor necrosis factor α (TNF-α), interleukin-1β (IL-1β), monocyte chemotactic protein-1 (MCP-1), intercellular adhesion molecule-1 (ICAM-1), nuclear factor-κB (NF-κB), and the phospho-p38 mitogen-activated protein kinase (p-p38-MAPK)/p38 MAPK ratio compared to control rats. LTF treatment significantly improved both retinal and pancreatic pathological injury, LTF treatment also inhibited inducible the p-p38 MAPK/p38 MAPK ratio and NF-κB activation and decreased the subsequent induction of the retinal expression of proinflammatory mediators TNF-α, IL-1β, MCP-1 and ICAM-1 compared to diabetic rats. LTF also exhibited a protective effect on islet function.

**Conclusions:**

LTF before the onset of DR can alleviate retinal pathological injury, LTF may play an anti-inflammatory role by inhibiting p38-MAPK and then inhibiting NF-κB pathway. But further studies are needed to confirm this conclusion.

*Trial registration* This is an animal experiment, trial registration is not necessary.

## Background

Diabetic retinopathy (DR) is one of the common and severe microvascular complications of diabetes mellitus (DM) and one of the leading causes of preventable blindness worldwide. The prevalence of DR increases with the duration of DM, and nearly all type 1 diabetic patients and more than 60% type 2 diabetic patients suffer from some degree of retinopathy in the 20 years after the oncet of DM [1–3]. Proliferative diabetic retinopathy is closely associated with vision loss, which is irreversible and seriously affects the quality of life. Early intervention is of great significance. Inflammation response contributes to the early development of DR [4–6]. Both an increase of inflammatory factors and leukocyte adhesion contribute to blood-retinal barrier (BRB) breakdown. In diabetic patients and diabetic animals, many proinflammatory factors are up-regulated in retinas, including tumor necrosis factor α (TNF-α), interleukin-1β (IL-1β), interleukin-6 (IL-6) and monocyte chemotactic protein-1 (MCP-1), etc., which are associated with vascular leakage. Additionally, studies have shown that hyperglycemia damages vasculature via inducing endothelial activation and pro-inflammatory phenotype of endothelial cells [7, 8], which is characterized by up regulation of cell surface adhesion molecules such as intercellular cell adhesion molecule-1(ICAM-1) and vascular cell adhesion molecule-1 (VCAM-1). These molecules cause the adhesion and results in the recruitment of leukocyte across endothelium, leading to retinal vascular inflammation and endothelial cell death, which ultimately induces DR [7–9]. Controlling inflammation can help reduce the overall severity of retinopathy and macular edema and restore the BRB [10]. Additionally, persistent hyperglycemia impairs the function of pancreatic beta cells and eventually leads to apoptosis. The function and quantity of pancreatic beta cells are essential for blood sugar homeostasis [11, 12]. The Chinese medicine compound, Luo Tong formula (LTF), composed of Huangqi (*Radix Astragali seu Hedysari*), Danshen (*Radix Salviae Miltiorrhizae*), Sanqi (*Radix Notoginseng*), Shuizhi powder (*Hirudo*), and Dahuang (*Rhubarb*), possesses blood invigorating properties and collaterals unblocking properties. We have previously shown that preventive treatment with LTF before the onset of DR can alleviate or delay the process of DR in diabetic rats. LTF can also ameliorate injury due to oxygen free radicals and improve endothelial cell function [13, 14]. All herbs in LTF have anti-inflammatory properties and have been found to inhibit inflammation by downregulating nuclear factor-κB (NF-κB) and p38 mitogen-activated protein kinase (p38-MAPK) signaling pathways [15–18]. However, the anti-inflammatory effects of LTF on DR are yet to be elucidated. In this study, we aimed to explore the mechanisms underlying the LTF modulation of retinal inflammation through p38-MAPK/NF-κB signaling pathways, providing new scientific evidence for complementary and alternative therapies.

## Materials and methods

### Standardization of Luo Tong formula

Medicine material crude slices of LTF adopted for this study were provided by Kangmei Pharmaceutical Co., Ltd. after quality assessment, and all herbs were from the same batch. Decoctions were prepared by the Guang`anmen Hospital, China Academy of Chinese Medical Sciences, according to standard operating procedures. LTFwas composed of Huangqi (*Radix Astragali seu Hedysari*), Danshen (*Radix Salviae Miltiorrhizae*), Sanqi (*Radix Notoginseng*), Shuizhi powder (*Hirudo*), and Dahuang (*Rhubarb*), in the ratio of 15:10:3:2:1. The major compounds in LTF were identified using high-performance liquid chromatography (HPLC; Waters 2695 HPLC system; Waters, CA, USA). A Luna^®^ Omega Polar C18 analytical column (250 × 4.6 mm, 3.0 μm; Phenomenex, CA, USA) with a mobile phase that contained acetonitrile (A) and -0.2% phosphoric acid acid in water (B) was used. The mobile phase gradient elution was programmed as follows: 23% A (0–10 min), 23–34% A (10–20 min), 34% A (20–31 min), 34–75% A (31–36 min) and 75% (36–60 min); 77% B (0–10 min), 77–66% B (10–20 min), 66% B (20–31 min), 66–25% B (31–36 min) and 25% (36–60 min). The column temperature was maintained at 35 ℃, the flow rate was set at 0.5 mL/min, and a detection wavelength of 203 nm was used. LTF was dissolved in double distilled water containing 0.05% dimethylsulfoxide (DMSO). The solution was centrifuged, filtered and disinfected using a syringe filter (specification: 13 mm nylon filter, 0.45 µm, 100 pcs/pack), and preserved at − 20 ℃ for further experimentation.

### Animal model and treatments

The study was approved by the Ethics Committee on Laboratory Animal Management of Guang’ anmen Hospital of China Academy of Chinese Medical Sciences. We abided by guidelines about animal use from the Association for Research in Vision and Ophthalmology (ARVO) throughout the study. Male 6-week-old Sprague–Dawley (SD) rats were purchased from Vital River Laboratory Animal Technology Co., Ltd. (Beijing, China) and acclimated for 1 week. Animals were maintained under a 12-h light/12-h dark cycle at 22 °C in cages, and the litter was kept dry. The animals had free access to food and water. Diabetes was induced by an intraperitoneal injection of streptozotocin (65 mg/kg, in 10 mmol/l citrate buffer, pH 4.5) in SD rats. Age-matched control rats received an equal volume of vehicle. Two days after the streptozotocin (STZ) injection, rats with a blood glucose level higher than 250 mg/dl were considered as diabetic [19]. The animals were divided into four groups as follows: (1) normal control rats (NC; n = 40), (2) STZ-induced diabetic rats (DM; n = 40); (3) STZ-induced diabetic rats treated with LTF (DM + LTF; 42 ml/kg, n = 40) and (4) STZ-induced diabetic rats treated with calcium dobesilate (DM + CaD; 104 mg/kg, n = 40). The doses of LTF and CaD were based on the daily dose commonly prescribed in humans.

### Examination of blood glucose

Rat tail-vein blood glucose at the different time points was measured using OneTouch Ultra test strips (Shen Zhen, China). At 12 weeks, all rats were anesthetized and euthanized, and blood samples were collected. Blood serum glucose (n = 6/group) was measured using an automatic biochemical analyzer (OLYMPUS-640, Tokyo, Japan).

### Light microscopic observations

Freshly removed eyeballs were fixed in 4% paraformaldehyde and embedded in paraffin. Sections were stained with hematoxylin–eosin (HE) and periodic acid-Schiff (PAS) staining for light microscopic observation. HE staining was used to analyze pathological changes in retinas and retinal digest preparations, and PAS staining was used to analyze pathological changes in retinal capillaries and for morphological quantification. Ten tangent retinal areas were randomly selected for observation, and images were taken and analyzed using Image-Pro Plus software (Media Cybernetics, America) to quantify endothelial cells and pericytes.

### Apoptosis assay

Rat retinal cell apoptosis was detected by TUNEL assay. Paraffin sections of retinas were dewaxed, diluted with distilled water, fixed in acetone for 8 min and washed three times with phosphate-buffered saline (PBS). The sections were blocked with 3% bovine serum albumin (BSA) for 30 min at room temperature. TUNEL reaction mixture (Roche, China) was added and incubated for 1 h at room temperature. Next, sections were washed three times with PBS, stained with DAPI for 1 min, and washed again. The mean optical densities of the sections were analyzed semiquantitatively with Image-Pro Plus analysis software (Media Cybernetics, America).

### Immunohistochemistry

Paraffin-embedded sections of retinas were dewaxed, diluted with distilled water, and blocked with 3% hydrogen peroxide for 15 min. Sections were then fixed in citrate under high pressure for 3 min, then incubated with primary antibodies against insulin (1:100 dilution) and glucagon (1:200 dilution) for 24 h at 4 °C. The sections were washed three times with PBS and incubated with secondary antibodies (goat anti-rabbit, 1:200; goat anti-mouse, 1:200) for 2 h at room temperature. Color Development Kits were used, with hematoxylin as a mild counterstain, the sections were dehydrated in a graded series of ethanol concentrations and sealed. The mean optical density of the sections was analyzed semiquantitatively with Image-Pro Plus analysis software (Media Cybernetics).

### Western blotting

After 12 weeks of treatment, rat retinas were harvested and washed with cold PBS, followed by incubation in RIPA buffer (Solarbio, China) on ice. Retinas were extracted and protein content was quantified using a BCA assay kit (Cwbiotech, China). After loading buffer was added, cell lysates were separated by 12% sodium dodecyl sulfate polyacrylamide gel electrophoresis (SDS-PAGE) and transferred onto polyvinylidene fluoride (PVDF) membranes (Millipore, USA). Membranes were blocked using Super Block T20 (TBS) buffer and incubated with primary antibodies (Cell Signaling Technology, USA) overnight at 4 °C, then washed 3 times with TBST [tris-buffered saline (TBS) and Tween 20]. Dilution ratios: mouse monoclonal antibodies against ICAM-1 (1:1000), rabbit monoclonal antibodies against TNF-α (1:1000), rabbit polyclonal antibodies against NF-κB (1:1000), rabbit polyclonal antibodies against p38-MAPK (1:1000). The membrane was then incubated with HRP-conjugated secondary antibodies (Jackson Immuno Research, USA) for 1 h, at the following dilution ratios: goat anti-rabbit, 1: 5000; goat anti-mouse, 1: 5000. Enhanced chemiluminescence (Millipore, USA) was used to visualize the bands and an image analyzer (Bio-Rad, USA) was employed for densitometric analysis. Protein expression was normalized to that of GAPDH (Abcam, USA).

### Real-time reverse transcription PCR assays (RT-PCR)

Total RNA was extracted using a Trizol Reagent Kit (GIBCO, New York, USA) according to the manufacturer’s instructions, and stored at − 40 °C until reverse transcription. Total RNA was reversely transcribed to first-strand cDNA, using a Reverse Transcription Kit; reaction conditions were as follows: 60 min at 42 °C and 10 min at 70 °C; cDNA was stored at − 40 °C until PCR. The primer sequences specific for IL-1β and MCP-1 used for the amplification are shown in Table 1. PCR reaction mixtures were added according to the recommended protocol of the SYBR Green Kit, and the DNA melting curves were acquired. PCR conditions were as follows: Stage 1: 1 cycle at 95 °C for 10 min; Stage 2: 40 cycles at 95 °C for 25 s, 55 °C for 25 s, 72 °C for 50 s; Stage 3:1 cycle at 72 °C for 8 min. The fluorescence signals were monitored, and three replicate tubes were prepared for each sample. Results were analyzed by using the 2^− △△CT^ method, and fold-changes over control were determined.

**Table 1:** Primers sequences used for real-time PCR

Genes	Forward primer	Reverse primer	Size (bp)
IL-1β	5′CCCTGCAGCTGGAGAGTGTGG3′	5′ACCAGTTGGGGAACTGTGCAGAC3′	126 bp
MCP-1	5′GGCCAGCCCAGAAACCAGCC3′	5′ACTGCATCTGGCTGAGACAGCAC3′	136 bp

### Statistical analysis

The experiments were performed at least three times, and all values are expressed as mean ± standard deviation (SD). Data were evaluated using one-way analysis of variance (ANOVA), the post hoc Test (Bonferroni methods) was performed following ANOVA. SPSS program (version 20.0; IBM Corp., Armonk, NY, USA) was used for the analyses. Values of *p *< 0.05 were considered statistically significant.

## Results

### HPLC analysis of LTF

**Fig. 1:**
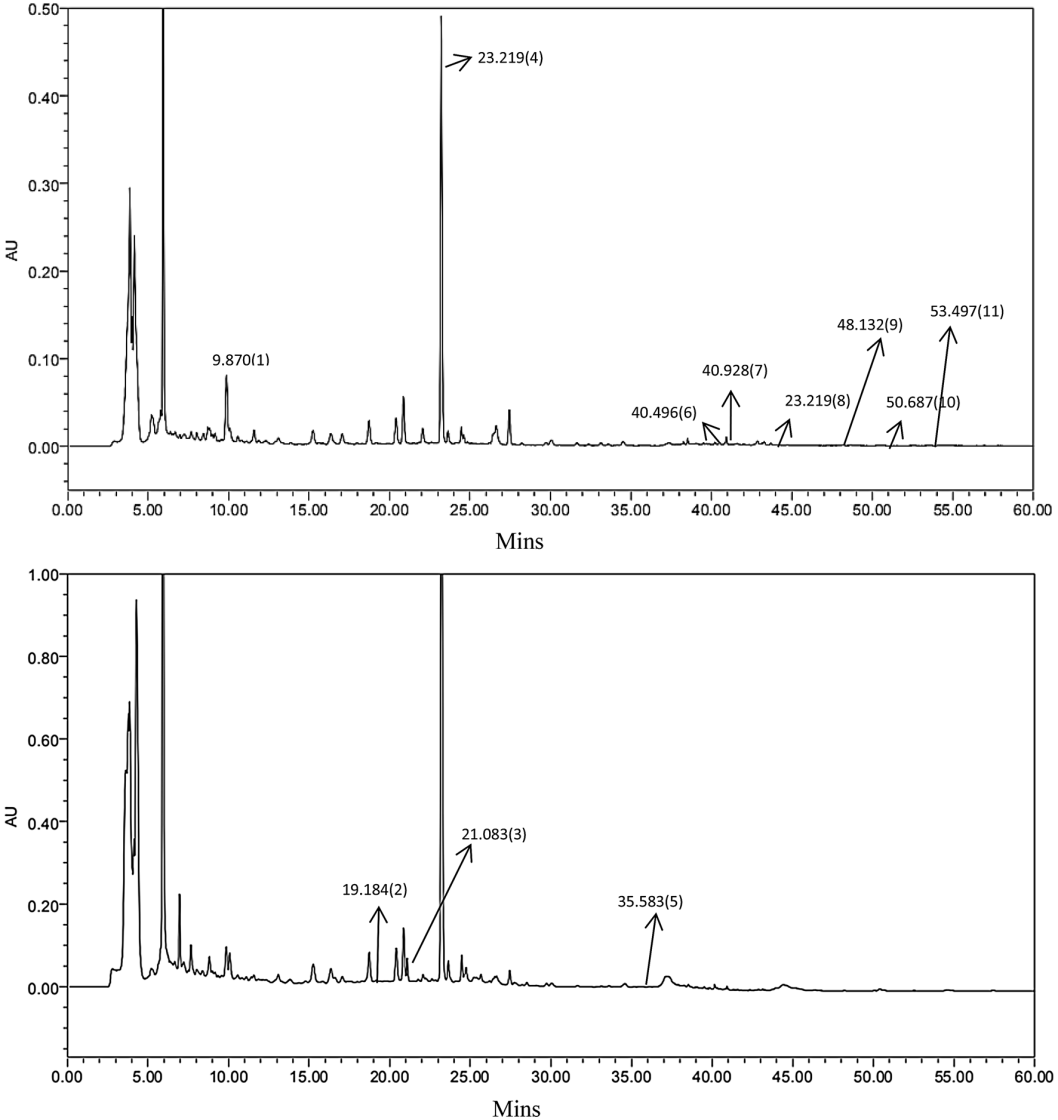
Chemical standardization of LTF by high performance liquid chromatography (HPLC) fingerprint analysis (254 nm and 210 nm). In the HPLC fingerprint at an absorbance of 254 nm, the peaks corresponding to Calycosin-7-O-β-d-glucoside (1); Notoginsenoside R1 (4); Ginsenoside Rb1(6); Aloe-emodin (7); Rhein (8); Emodin (9); Chrysophanol (10); Physcion and Tanshinone II A (11). In the HPLC fingerprint at an absorbance of 210 nm, the peaks corresponding to (2) Ginsenoside Rg1; (3) Salvianolic acid B and (5) Aloe-emodin

In order to standardise the chemical composition of the compound herbal medicine LTF, we performed HPLC fingerprint analysis. Figure 1 shows a typical HPLC fingerprint of LTF, in which the major peaks were identified by comparing both the retention times of both LTF and the reference standards. Notably, 11 compounds in LTF, viz. (1) Calycosin-7-O-β-D-glucoside, (2) Notoginsenoside R1, (3) Ginsenoside Rg1, (4) Salvianolic acid B, (5) Ginsenoside Rb1, (6) Aloe-emodin, (7) Rhein, (8) Emodin, (9) Chrysophanol, (10) Physcion, and (11) Tanshinone II A were properly identified (Fig. 1).

### Effects of LTF on body weight and blood glucose

**Fig. 2:**
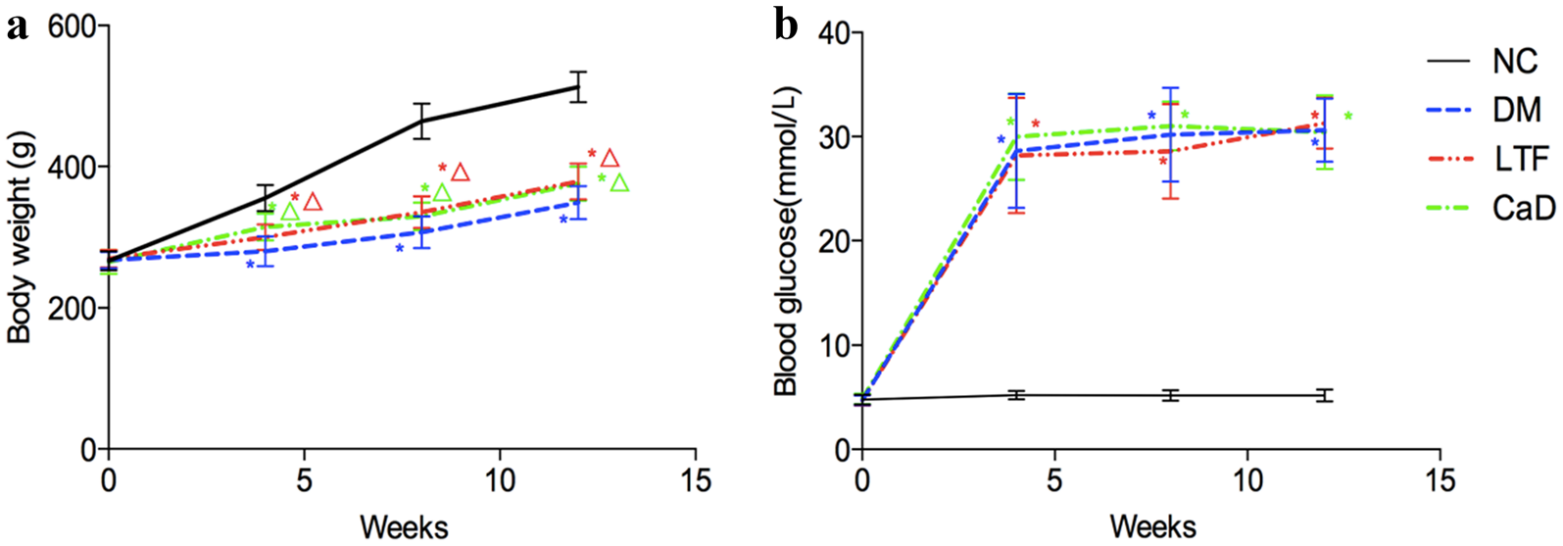
Effects of LTF and CaD on body weight (**a**) and blood glucose (**b**). (*p < 0.05: versus NC group; △ *p *< 0.05: versus DM group)

Hyperglycemia significantly lead to weight loss of rats. As shown in Fig. 2a, 4 weeks after STZ injection, the body weight of DM rats was markedly decreased compared to that of normal controls (NC) (*4* *weeks*: 279.76 ± 20.96 versus 355.25 ± 18.34; *8* *weeks*: 306.64 ± 22.39 versus 464.20 ± 25.22; *12* *weeks*: 348.69 ± 23.66 versus 512.80 ± 21.69, *P *<* 0.05*). However, compared with the DM group, LTF and CaD both attenuated HG-induced weight loss, and the body weight of LTF-treated rats was significantly increased to 299.51 ± 18.47, 335.20 ± 22.76, and 378.54 ± 25.47 at 4, 8, 12 weeks, respectively (*P *<* 0.05*); that of CaD-treated rats was significantly increased to 314.34 ± 18.91, 329.19 ± 19.35, and 375.62 ± 24.23 at 4, 8, 12 weeks, respectively (*P *<* 0.05*). Significant difference existed between LTF-treated groups and CaD-treated group at 4 weeks (*P *<* 0.05*), but LTF-treated groups failed to significantly increased the body weight at 8 week and 12 week compared with CaD-treated group (*P* > 0.05) (Fig. 2a).

At each time point, blood glucose levels in the DM group were dramatically higher than in the NC group (*4* *weeks*: 30.18 ± 4.49 versus 5.20 ± 0.50; *8* *weeks*: 30.61 ± 3.03 versus 5.20 ± 0.58; *12* *weeks*: 31.38 ± 2.20 versus 5.23 ± 0.51, *P *<* 0.05*, respectively), and compared with the DM group, the blood glucose levels of LTF-treated rats was significantly decreased to 28.58 ± 4.53 at 4 week (*P *<* 0.05*), significant difference existed between LTF-treated and CaD-treated groups (28.58 ± 4.53 versus 31.03 ± 2.35, *P *<* 0.05*) at 4 weeks. In LTF and CaD group, they were comparable to those of DM rats at 8 and 12 weeks (*P *>* 0.05*), and no significant difference existed between the two groups (*P *>* 0.05*) (Fig. 2b).

### Effects of LTF on the development of DR

**Fig. 3:**
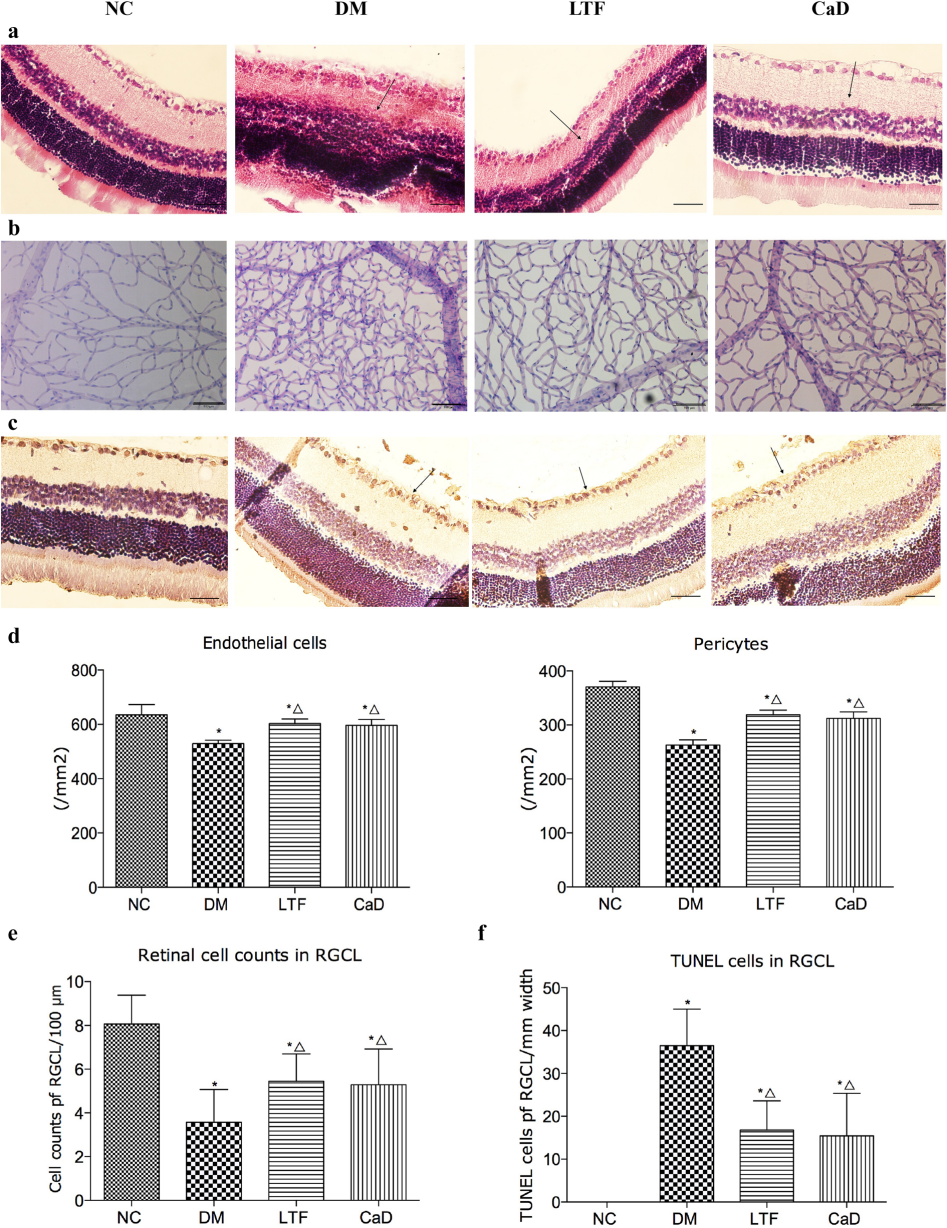
Effect of LTF and CaD on the retinal pathological injury. **a** HE staining of retina (n = 6); **b** PAS staining of retina (n = 6) (*p < 0.05: versus NC group; △ *p *< 0.05: versus DM group); **c** TUNEL staining in retina (n = 4; × 400); **d** measurement of endothelial cells and pericytes; **e** the respective quantitative analysis of Retinal cell counts of GCL; **f** measurement of TUNEL-positive cells. (*p < 0.05: versus NC group; △p < 0.05: versus DM group). Scale bar: 50 μm

Normal control rats showed a normal retinal morphology; all cell layers in the retina were clear and neatly arranged. In the DM group, the retinal tissue showed slight edema, the cell boundaries between each layer were ambiguous, and the ganglion cells were reduced. In the LTF and CaD groups, each layer of the retina was regularly arranged, and slight tissue edema but no telangiectasia was observed (Fig. 3a). Retina digest preparations showed that after 12 weeks of treatment, in the NC group the capillary network was arranged in an orderly, straight structure, and the vascular diameter was uniform. On the other hand, in the DM rats, the capillary network was disordered, and the basement membrane slightly thickened. Notably, in LTF and CaD rats, retinal digest preparations showed features similar to those of the control group. In particular, the distribution of capillaries was normal in these groups (Fig. 3b). The numbers of pericytes and endothelial cells were normal in the NC group. After 12 weeks of DM induced by STZ, the numbers of pericytes and endothelial cells began to decrease in the DM group (*P* < 0.05). LTF and CaD significantly reversed the number of pericytes and endothelial cells, thus inhibiting acellular capillary formation (*P* < 0.05) (Fig. 3d).

In consistent with the diabetes-mediated alterations of retinal structure, the results of TUNEL staining indicated that retinal apoptosis-positive staining was much stronger in the DM than in the NC group, and the apoptosis-positive staining mainly assembled in the layers of ganglion cell and nerve fiber. After treatment with LTF and CaD, apoptotic staining was less pronounced than in DM retinas (Fig. 3c). Moreover, there are significantly reduced cell number of retinal ganglion cell layer (RGCL) and increased the numbers of TUNEL-positive cells in the RGCL of the diabetic retina (Retinal cell counts: 3.57 ± 1.50 versus 8.06 ± 1.32; TUNEL-positive cells: 36.46 ± 8.55 versus 0; *P *<* 0.0*5, respectively) compared with to NC retina. Meantime, treatment with LTF was able to significantly prevent the reduction of retinal cells (5.45 ± 1.25 versus 3.57 ± 1.50, *P *<* 0.05*) and decrease retinal cell apoptosis (16.75 ± 6.84 versus 36.46 ± 8.55, *P *<* 0.05*) compared with that of the diabetic retina. CaD treatments also markedly attenuated the reduction of retinal cells and the increase of TUNEL-positive cells compared to that of diabetic (Retinal cell counts: 5.29 ± 1.63 versus 3.57 ± 1.50; TUNEL-positive cells: 15.44 ± 9.90 versus 36.46 ± 8.55; *P *<* 0.0*5, respectively) (Fig. 3e, f). The proportion of retinal cell counts and TUNEL-positive cells in LTF-treated and CaD-treated groups indicated to be no significantly difference (*P* > 0.05). Taken together, the results suggested that administration of LTF to rats can correct the diabetes-induced retinal structural abnormalities and inhibit the development of DR.

### Effects of LTF on the expression of inflammatory mediators

**Fig. 4:**
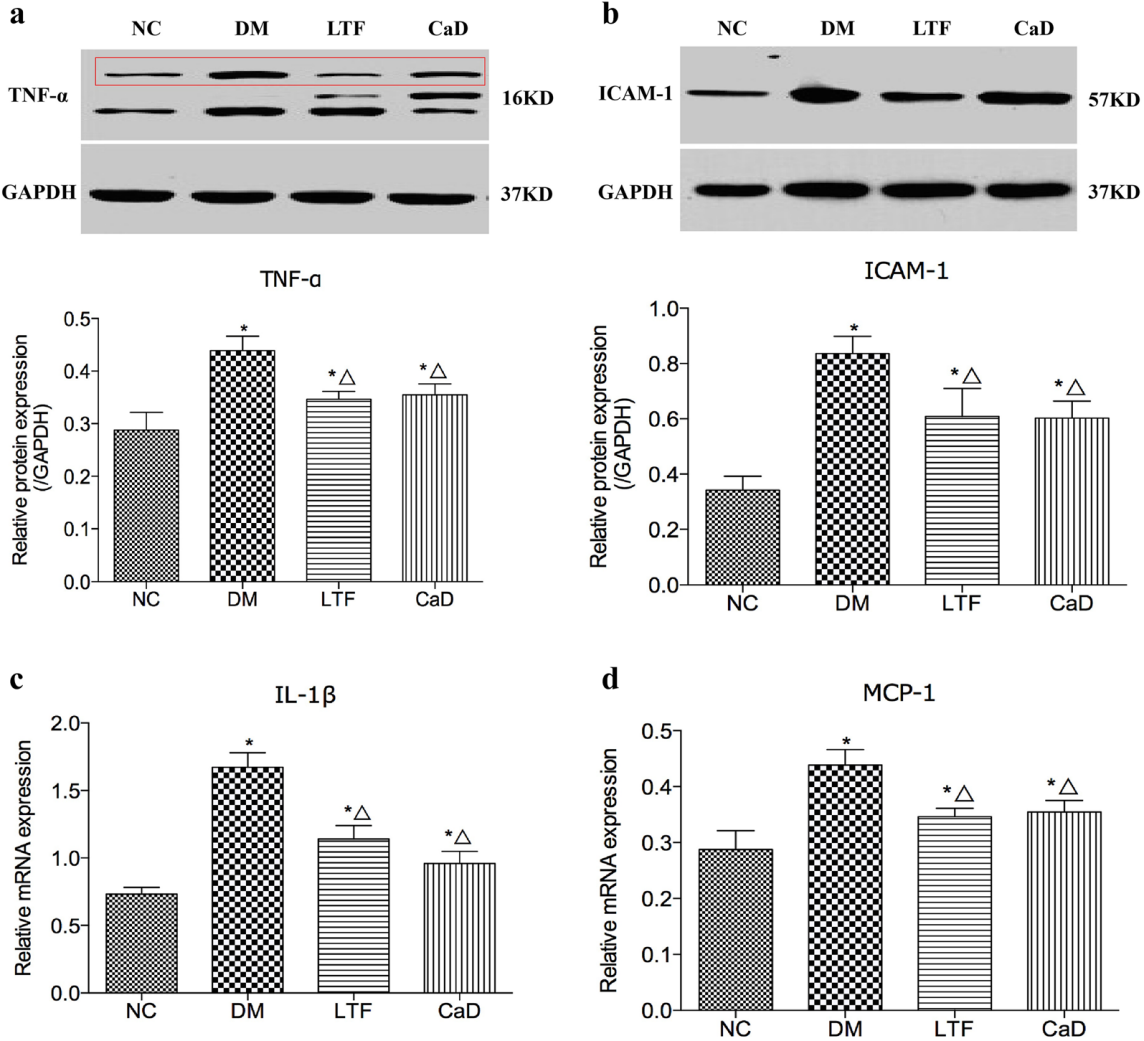
Effect of LTF and CaD on the expression of TNF-α, ICAM-1, IL-1β, and MCP-1; **a**–**b**: western blot and quantitative measurement of retinal TNF-α and ICAM-1(n = 4); (**c**–**d**): real-time PCR analysis of retinal IL-1β and MCP-1. (*p < 0.05: versus NC group; △p < 0.05: versus DM group)

To determine the mechanisms underlying the LTF-mediated inhibition of DR, protein and mRNA expression levels of TNF-α, ICAM-1, IL-1β, and MCP-1 were determined by western blot and real-time PCR. As presented in Fig. 4, TNF-α protein expression levels were markedly increased in the DM group compared to those in the NG group (0.44 ± 0.07 versus 0.29 ± 0.09, *P *<* 0.05*). TNF-α upregulation was substantially attenuated in the LTF and CaD groups compared to the DM group (LTF: 0.35 ± 0.03 versus 0.44 ± 0.07, *P* < 0.05; CaD: 0.35 ± 0.05 versus 0.44 ± 0.07, *P* < 0.05), LTF significantly decreased TNF-α levels compared with CaD (*P* < 0.05). The statistical data of TNF-α was *F *= 5.605 and *P *= 0.006. ICAM-1 expression was also markedly increased in the DM group when compared to that in the NG group (ICAM-1: 0.84 ± 0.15 versus 0.34 ± 0.14, *P* < 0.05), and ICAM-1 protein expression was also significantly lower in the LTF and CaD groups compared to the DM group (0.61 ± 0.28 versus 0.84 ± 0.15 and 0.60 ± 0.15 versus 0.84 ± 0.15, respectively, *P* < 0.05 for LTF group and CaD group), but no significant difference existed between LTF and CaD groups (*P* > 0.05). The statistical data of ICAM-1 was *F *= 7.334 and *P *= 0.001. IL-1β gene expression levels were markedly enhanced in the DM rats compared to controls (1.67 ± 0.27 versus 0.73 ± 0.12, *P *<* 0.05*). However, the increase in IL-1β gene expression was markedly attenuated in LTF and CaD compared to DM rats (1.14 ± 0.24 versus 1.67 ± 0.27, *P* < 0.05 and CaD: 0.96 ± 0.22 versus 1.67 ± 0.27, *P* < 0.05, respectively), however, no obvious difference in IL-1β expression was observed between the groups treated with LTF and CaD (*P* > 0.05). The statistical data of IL-1β was *F *= 20.176 and *P *= 0.000. Similar results were obtained for MCP-1 gene expression, MCP-1 expression was significantly upregulated in the DM group (2.06 ± 0.57 versus 0.56 ± 0.12, *P *< 0.05), and this expression was dramatically reversed by LTF and CaD treatment (1.35 ± 0.40 versus 2.06 ± 0.57, *P* < 0.05 and 1.07 ± 0.41 versus 2.06 ± 0.57, *P* < 0.05, respectively), but no significant difference existed between LTF and CaD groups (*P* > 0.05). The statistical data of MCP-1 was *F *= 14.004 and *P *= 0.000. Therefore, LTF prevented the development of DR probably by downregulating TNF-α expression and ICAM-1 protein expression and downregulating IL-1β and MCP-1 gene expression.

### Effects of LTF on NF-κB (p65) and p38-MAPK pathways

**Fig. 5:**
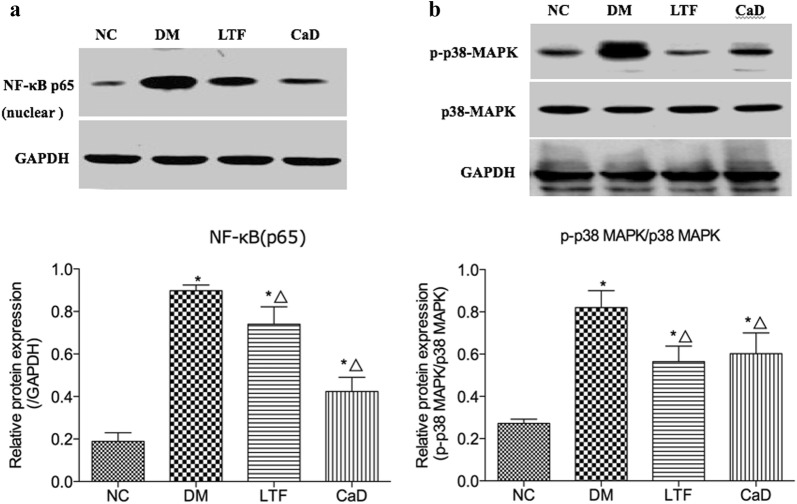
Effect of LTF and CaD on NF-κB p65 and p38-MAPK pathways. **a** Western blot and quantitative measurement of retinal NF-κB(p65); Lane loading was normalized by reblotting with GAPDH. **b** Western blot and quantitative measurement of p38-MAPK and phosphorylated p38-MAPK. Lane loading was normalized by reblotting with GAPDH. The levels were expressed as p- p38-MAPK/p38-MAPK and normalized relative to control group (n = 4). (*p < 0.05: versus NC group; △p < 0.05: versus DM group)

To further investigate the molecular mechanisms underlying the anti-inflammatory effect of LTF, western blot analysis was performed to determine the potential role of NF-B p65 and p38MAPK activation in the retina of diabetic rats. There was significant increase in the concentration of nuclear NF-κB p65 in retinal tissues of STZ induced diabetic rats than control group of rats (0.90 ± 0.07 versus 0.19 ± 0.12, *P *<* 0.05*). However, treatment with LTF and CaD significantly decreases its concentration in the retinal tissues compared to STZ-diabetic rats. (LTF: 0.74 ± 0.20 versus 0.90 ± 0.07, *P* < 0.05; CaD: 0.44 ± 0.19 versus 0.90 ± 0.07, *P* < *0.05*, respectively). CaD significantly decreased NF-κB p65 levels compared with LTF (*P* < 0.05). The statistical data of NF-κB p65 was *F *= 35.952 and *P *= 0.000 (Fig. 5a). P38-MAPK pathway was found to be activated in diabetic retinas. The activation of p38 MAPK, which was evaluated by the ratio of phospho-p38 MAPK/p38 MAPK, significantly increased in the retinas of diabetic rats (0.82 ± 0.20 versus 0.27 ± 0.05, *P* < 0.05). Such increase was dramatically reversed by LTF (0.56 ± 0.19 versus 0.82 ± 0.20, *P* < 0.05). Treatment with CaD also significantly down-regulated the p-p38 MAPK/p38 MAPK in the retinas versus that in the DM group (CaD: 0.60 ± 0.23 versus 0.82 ± 0.20, *P* < 0.05). However, no significant difference existed between LTF-treated groups and CaD-treated group (*P* > 0.05). The statistical data of p38-MAPK was *F *= 8.953 and *P *= 0.001 (Fig. 5b).

### Effects of LTF on panceratic insulin and glucagon

**Fig. 6:**
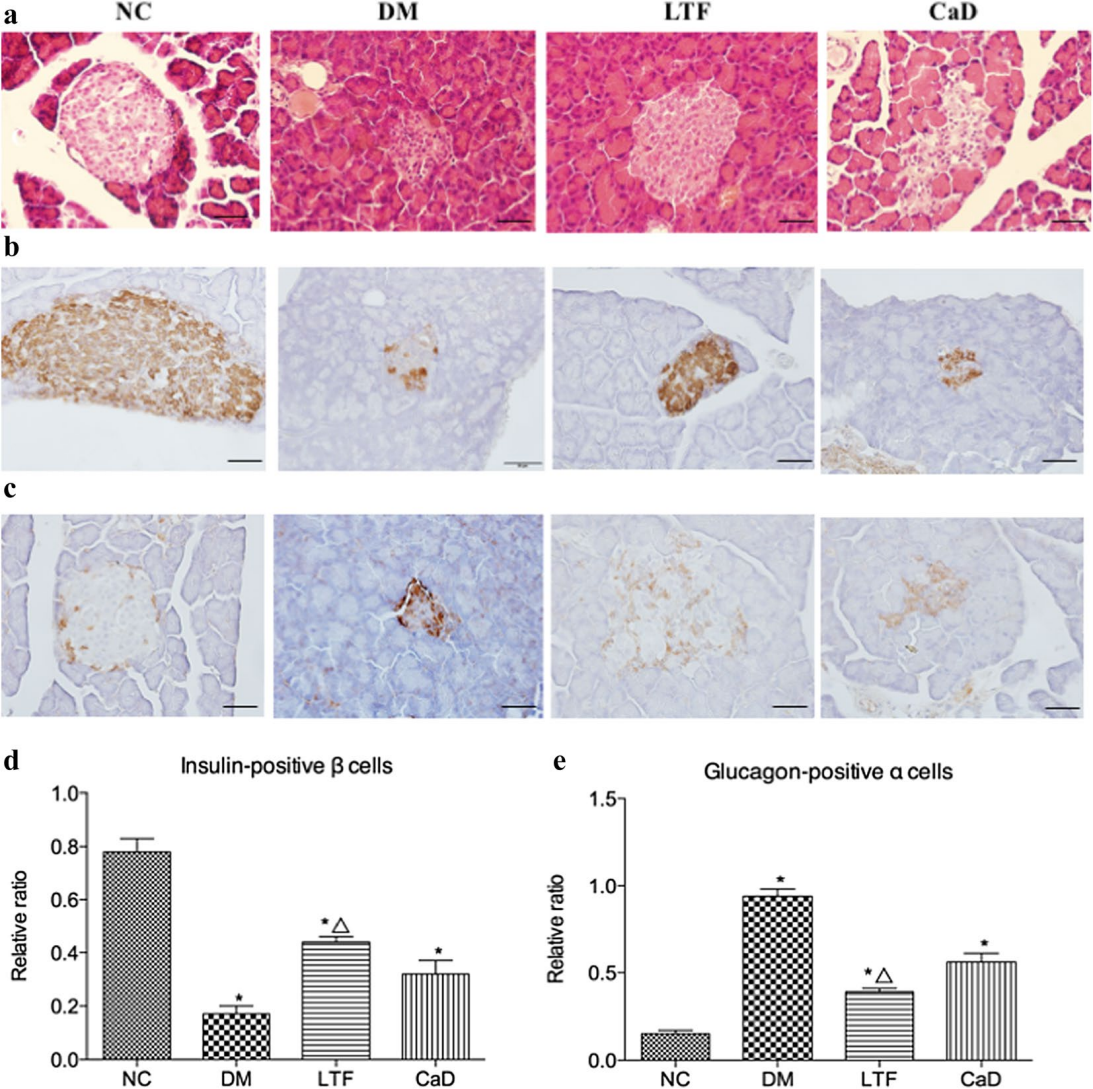
Effect of LTF and CaD on the islet function. **a** HE staining of pancreas (n = 6); **b**, **c** Immunostaining for pancreatic insulin and glucagon (n = 4; × 400); **d**, **e** quantitative analysis of pancreatic insulin and glucagon (n = 4; × 400). (*p < 0.05: versus NC group; △ *p *< 0.05: versus DM group). Scale bar: 50 μm

HE staining indicated that in the NC group, the morphology of the islets was round or oval cell mass, there was the high number of pancreatic cells, and the boundary was clear, while the morphology of the islets in the DM group was irregular, and the number of pancreatic cells decreased and the distribution was sparse; In the LTF and CaD group, the morphology of the islets was less regular, the number of pancreatic cells decreased, and the boundaries of the exocrine glands were relatively clear, and the arrangement of the pancreas was more regular compared with DM group (Fig. 6a). Immunohistochemistry indicated a lower number of insulin-positive β cells in DM rats compared to controls. The expression levels of insulin-positive β cells in the pancreas were markedly down-regulated in the DM group compared with that in the NC group (0.17 ± 0.03 versus 0.78 ± 0.05, *P *<* 0.05*). Treatment with LTF resulted in a less pronounced decline of the insulin-positive staining similar to that of DM rats (0.44 ± 0.02 versus 0.17 ± 0.03, *P *<* 0.05*). CaD failed to exert a protective effect on pancreatic cells comparable to that observed in LTF animals (0.32 ± 0.05 versus 0.17 ± 0.03, *P *>* 0.05*) (Fig. 6b–d). Besides, a more number of glucagon-positive α cells was captured in DM rats compared to controls. The expression levels of glucagon-positive α cells in the pancreas were markedly up-regulated in the DM group compared with that in the NC group (0.94 ± 0.04 versus 0.15 ± 0.02, *P *<* 0.05*). Treatment with LTF reduced the expression levels of glucagon-positive α cells (0.39 ± 0.02 versus 0.94 ± 0.04, *P *<* 0.05*). CaD failed to exert a protective effect on pancreatic cells comparable to that observed in LTF animals (0.56 ± 0.05 versus 0.94 ± 0.04, *P *>* 0.05*) (Fig. 6c–e).

## Discussion

LTF, modified based on the Di Dang Decoction (*Synopsis Golden Chamber*), is a commonly used traditional Chinese medicine prescribed for treating diabetic microvascular complications [20, 21]. In this study, we showed that STZ-induced diabetes resulted in DR, and 12 weeks of LTF treatment ameliorated retinal damage, delayed DR occurrence, and significantly decreased the expression of TNF-α, IL-1β, MCP-1, and ICAM-1. The latter effect was probably associated with decreased retinal inflammation in the diabetic rats through modulation of the NF-κB(p65) and p38-MAPK pathways. In addition, 12 weeks of LTF treatment helped improve the pathological damage to pancreas, increased insulin expression in pancreatic beta cells, and downregulated the expression of glucagon in pancreatic α cells.

Pro-inflammatory cytokines/chemokines play an essential role in its pathogenesis. The published researches indicated that anti-inflammatory medication could delay early DR [4–6]. Huangqi, Danshen, Sanqi, Shuizhi powder, and Dahuang have been shown to inhibit the expression of pro-inflammatory mediators or chemokines such as IL-1β, IL-6, TNF-α, ICAM-1 and MCP-1, as well as signaling cascades such as the NF-*κ*B pathway [18, 22, 23]. TNF-α is an important pro-inflammatory cytokine that is involved in numerous inflammatory pathologies. Upregulation of TNF-α has been previously detected in the retinas of diabetic rats and DR patients [24]. Inhibition of TNF-α was demonstrate to suppress NF-κB activation, leukostasis, and BRB breakdown [25]. Interleukin-1β has effects of increasing of vascular permeability, promoting the retinal cell apoptosis and leukocyte adhesion. IL-1β is mainly mediated by NF-κB activation, and increased expression of IL-1β may contribute to increased production of reactive oxygen species and further NF-κB activation [26]. MCP-1 is a potent chemotactic factor for monocytes, macrophages, and T lymphocytes, which can help to promote the expression of superoxide and other mediators. MCP-1 can chemotactically aggregate monocytes, which leads to capillary obstruction, vascular leakage and absence of perfusion. Increased expression of MCP-1 is also implicated in the pathogenesis of DR [27]. Here, we confirmed that LTF suppressed the expression of TNF-α, IL-1β, and MCP-1, which may contribute to the beneficial effects of LTF treatment. Adhesion of leukocytes to the retinal endothelium is increased in diabetic rats (known as leukostasis) and contributes to early DR, inducing the formation of acellular capillaries, vascular endothelial dysfunction, BRB breakdown, and increased vascular permeability. Our results indicated that LTF suppressed the expression of ICAM-1.

NF-κB activation can induce the release of some pro-inflammatory cytokines, such as IL-1β, IL-6, MCP-1 and TNF-α, which have been considered as central mediators of inflammatory processes, and NF-κB inhibition in diabetic retinas prevents the enhancement of ICAM-1, leukocyte adhesion and BRB leakage [24]. In this study, we found that hyperglycemia induced the increase of NF-κB (p65) expression levels in rat retina, indicating that hyperglycemia can activate NF-κB pathway. After LTF treatment, the expression of NF-κB (p65) was decreased, which suggested that LTF might exert a protective role in rat retina through NF-κB pathway. Under hyperglycemic condition, p38MAPK is activated in the retina and involved in inflammatory response, endoplasmic reticulum stress and apoptosis. Hyperglycemia induced activation of the MAPK family, phosphorylation of related proteins and mediation of inflammatory response [28, 29]. Blocking p38-MAPK may be involved in alleviating inflammation. Previous studies have shown that herbal medicine promoting blood circulation and preventing vascular stasis relieves retinal pathological damage in diabetic mice, possibly as a consequence of reduced p38-MAPK phosphorylation [30]. Consistently with this previous study, result indicated that LTF markedly downregulated the expression of p38-MAPK, suggested that the protective effect of LTF was related to the inhibition of p38-MAPK activation. NF-κB is closely related to p38-MAPK, both of which gather together in the downstream of p38-MAPK. Activated p38-MAPK activation could activate NF-κB by promoting the expression of inflammatory cytokines, which is an important downstream site of p38-MAPK signal pathway. If the expression of p38-MAPK is inhibited, the activation of NF-κB will also be affected [31]. This study found that both p38MAPK and NF-κB pathways could be inhibited by LTF, suggesting that LTF play an anti-inflammatory role by inhibiting p38-MAPK and then inhibiting NF-κB pathway. But further studies are needed to confirm this conclusion.

In addition, LTF apparently promoted the expression of insulin while suppressing that of glucagon, suggesting a function in islet protection. However, this study was not able to conclusively prove this. The efficacy of Chinese herbal medicines, such as extracts, monomers, and compounds, in protecting islet beta cells has been gradually recognized, and may contribute to the prevention and treatment of DM and related complications. This protective action towards islet beta cells may result in a plethora of effects including increased cell proliferation, reduced apoptosis, attenuation of oxidative stress, promotion of insulin resistance, improvement of islet microcirculation, and regulation of autophagy, among others [32].

This study has several limitations. First, the molecular targets of LTF may not dependent on the NF-κB and/or the p38-MAPK signalling pathway, other mechanism should not exclude. We will continue to further explore the mechanism of LTF. Second, additional experiments in human retinal microvascular endothelial cells are needed to ascertain DR prevention by LTF. Third, leukocyte adhesion was not addressed in vitro.

## Conclusion

In summary, our findings show that preventive treatment with LTF before the onset of DR can alleviate retinal pathological injury and decrease the expression of pro-inflammatory factors. LTF effects were correlated with the inhibition of NF-κB and p38-MAPK pathways. Additionally, preliminary evidence regarding the potential of LTF as an islet protective agent was provided.

## Data Availability

The datasets used or analyzed during the study are available from the corresponding author on reasonable request.

## Abbreviations

ANOVA: one-way analysis of variance
ARVO: Association for Research in Vision and Ophthalmology
BRB: blood-retinal barrier
BSA: bovine serum albumin
CaD: calcium dobesilate
DM: diabetes mellitus
DR: diabetic retinopathy
HE: hematoxylin–eosin
HPLC: high-performance liquid chromatography
IL-1β: interleukin-1β
ICAM-1: intercellular adhesion molecule-1
LTF: Luo Tong formula
MCP-1: monocyte chemotactic protein-1
NC: normal control
NF-κB: nuclear factor-κB
P38-MAPK: p38 mitogen-activated protein kinase
PAS: periodic acid-schiff
PBS: phosphate-buffered saline
PVDF: polyvinylidene fluoride
SD: standard deviation
SDS-PAGE: sodium dodecylsulfate polyacrylamide gel electrophoresis
TNF-α: tumor necrosis factor α
VCAM-1: vascular cell adhesion molecule-1
